# Charge Transfer Mechanism in Guanine-Based Self-Assembled Monolayers on a Gold Surface

**DOI:** 10.1021/acs.langmuir.4c01512

**Published:** 2024-07-10

**Authors:** Jesús Lucia-Tamudo, Juan J. Nogueira, Sergio Díaz-Tendero

**Affiliations:** † Department of Chemistry, Universidad Autónoma de Madrid, 28049 Madrid, Spain; ‡ Institute for Advanced Research in Chemistry (IAdChem), Universidad Autónoma de Madrid, 28049 Madrid, Spain; ¶ Condensed Matter Physics Center (IFIMAC), Universidad Autónoma de Madrid, 28049 Madrid, Spain

## Abstract

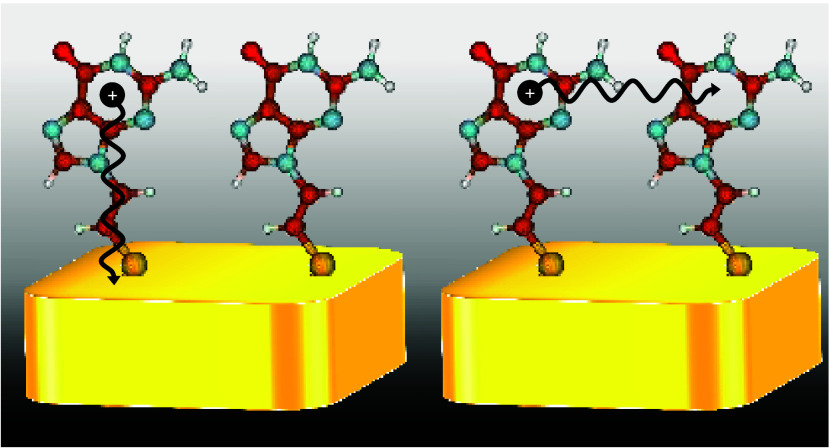

In this work, we have theoretically determined the one-electron oxidation potentials and charge transfer mechanisms in complex systems based on a self-assembled monolayer of guanine molecules adsorbed on a gold surface through different organic linkers. Classical molecular dynamics simulations were carried out to sample the conformational space of both the neutral and the cationic species. Thus, the redox potentials were determined for the ensembles of geometries through multiscale quantum-mechanics/molecular-mechanics/continuum solvation model calculations in the framework of the Marcus theory and in combination with an additive scheme previously developed. In this context, conformational sampling, description of the environment, and effects caused by the linker have been considered. Applying this methodology, we unravel the phenomena of electric current transport by evaluating the different stages in which charge transfer could occur. The results revealed how the positive charge migrates from the organic layer to the gold surface. Specifically, the transport mechanism seems to take place mainly along a single ligand and driven with the help of the electrostatic interactions of the surrounding molecules. Aside, several self-assembled monolayers with different linkers have been analyzed to understand how the nature of that moiety can tune the redox properties and the efficiency of the transport. We have found that the conjugation between the guanine and the linker, at the same time conjugated to the gold surface, gives rise to a more efficient transport. In conclusion, the established computational protocol sheds light on the mechanism behind charge transport in electrochemical DNA-based biosensor nanodevices.

## Introduction

In the last few decades, there has been a considerable increase in the applications of DNA. Despite the fact that DNA is primarily a biochemical macromolecule used for storing the genetic code of an organism, its transversal applications are numerous.^1^ In this article we take advantage of two of them. The first one is the use of DNA strands as nanowires,^2,3^ which has been extensively studied in recent years. DNA has the ability to transport electric charge along its strand, making it a suitable macromolecule for conduction purposes. Consequently, DNA can be anchored to an electrode or other device that transfers a hole or an electron to the DNA strand so that it can migrate along its nucleobases. On the other hand, an ensemble of DNA strands can also be adsorbed onto a metallic surface to form a self-assembly monolayer (SAM),^4−7^ which can be used for molecular detection.^8,9^ This is typically known as DNA-based biosensors.^10−13^

In general terms, a sensor is a device that can qualitatively or quantitatively detect the presence of a chemical species of interest in a sample. It usually consists of a receptor, which traps the analyte, a transducer, which converts the nature of the chemical signal into a measurable one, and a signal processing device, which measures the transformed signal. Specifically, a biosensor is a type of sensor whose receptor is constituted by a biomolecule. These particular sensors are becoming increasingly popular in many fields, such as health services,^14−18^ control assurance,^19−22^ or environment,^23,24^ due to the vast number of gadgets that can be designed.^25,26^ In addition, the most commonly used biosensors employ electrochemical techniques in the detection task,^27,28^ which are typically based on the formation and/or destruction of one or more electrochemical species.^29,30^ This means that the electrochemical species interacts with the bioreceptor transferring electrons and following a reduction–oxidation type of reaction.

For a successful design of a DNA-based biosensor, there are several important considerations to be addressed. First, the surface can induce conformational changes in the DNA structure that can affect the efficiency of the electron transfer process and the sensitivity of the biosensor. To avoid these issues, it is important to carefully choose the immobilization conditions, to ensure that the DNA retains its native structure and remains stable on the surface.^31^ Various methodologies can be employed for this purpose,^32^ but the most efficient approach involves anchoring the DNA strand using a linker, typically based on a functionalized small thiol. It has been demonstrated that thiolated organic molecules strongly adsorb onto gold surfaces due to the favorable Au–S interaction.^33,34^

**Figure 1:**
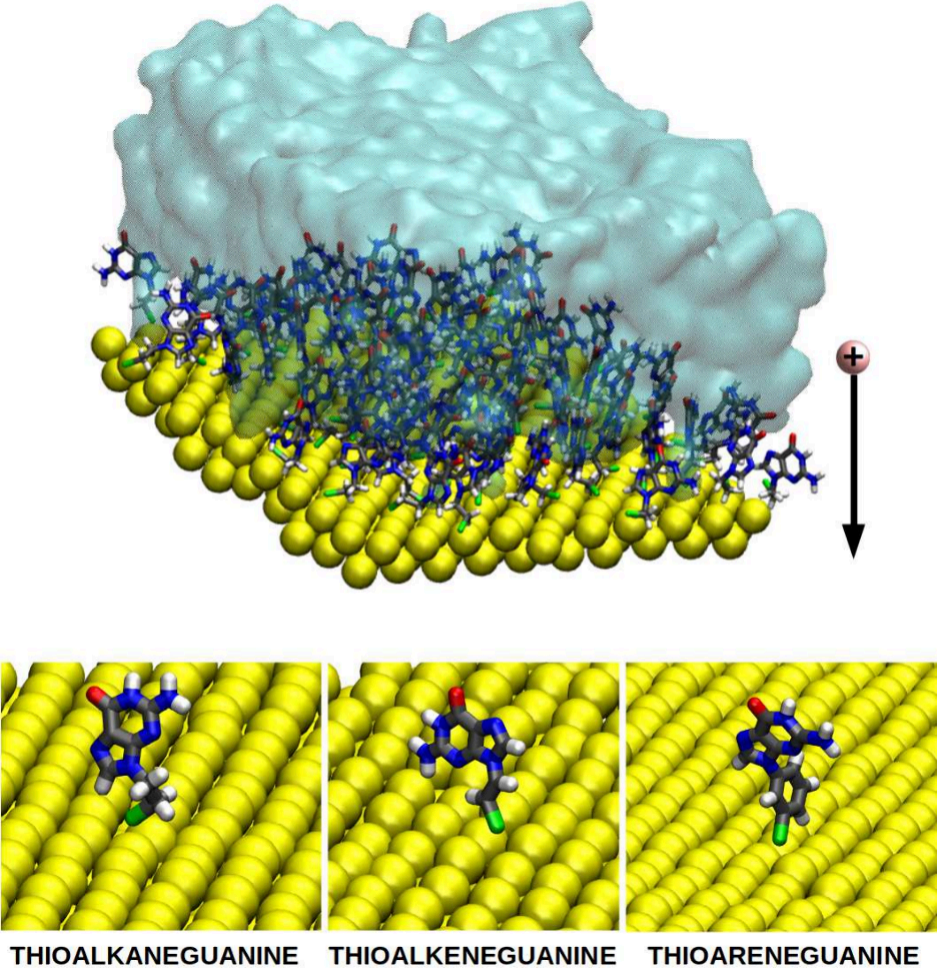
Scheme representing the SAM with the charge transfer process under study. The three ligands, composed by the guanine and the linkers, are represented separately. Color code: C atoms in gray, N in blue, O in red, H in white, S in green, Au in yellow and the cyan surface represents the water solvent.

Furthermore, in electrochemical biosensors, both the DNA strand and the substrate exchange a hole or an electron, so it is essential to gain insight into the operating mechanism that allows such current exchange, as well as the redox properties of the system at the different stages of the process. In particular, redox properties such as the one-electron oxidation potential and how the charge is delocalized along the SAM are crucial factors to be considered. In a DNA strand, it has been shown that electron transfer mainly occurs between nucleobases in water, making the determination of the redox properties of these moieties of paramount importance.^35−46^ From these results, it can be observed that guanine is more susceptible to oxidation. In a previous study, we elucidated the one-electron oxidation potential of a simplified model of a DNA-based biosensor based on a SAM composed of guanine residues, along with a complete protocol for accurately calculating this property within these systems.^47^ The results showed that the reducer character of the nucleobase increases when it is placed on a SAM, leading to a more effective biosensor. In this manuscript, we use the proposed methodology and computational protocol to obtain useful chemical information concerning mechanistic details of the charge transfer process in the organic-metal interface region; thus providing information on the functioning at the molecular level of DNA-based electrochemical biosensors. In particular, we examine three different examples of a simplified model of a DNA-based biosensor, in which guanine molecules are anchored to a Au(100) surface forming a SAM. The immobilization technique previously mentioned has been utilized, and we consider that the nucleic residue is assembled onto the surface through three small thiolated linkers: an alkane, an alkene and an arene (see Figure 1). The main aim of this study is to determine the various manners in which a positive charge can be transferred when it reaches the nucleotide of a DNA strand located close to the surface. Thus, we are referring to the last step of the biosensing mechanism: the charge transfer between the DNA strand and the metallic substrate. However, other previous steps such as the transport along the DNA strands and the factors that affect to this phenomenon were deeply analyzed in some previous works.^48,49^ A comparative analysis of the delocalization of the positive charge has been also conducted, which has allowed us to discern charge transfer mechanisms at the interface.

## Materials and Methods

### Computational Details

Due to the complexity of the system under study, formed by a self-assembled monolayer (SAM) of organic molecules adsorbed on a gold surface, we apply a dynamical protocol to populate an ensemble of conformations along the potential energy surface using classical molecular dynamics (MD). Properties are then computed by averaging the value obtained over all populated conformations by means of a multilayer QM/MM/COSMO scheme. In this work, oxidation potentials are computed using the Marcus theory^50−55^ (see details on the methods in the Supporting Information). The QM/MM/Continuum calculations for both the neutral and cationic forms of the SAMs, were carried out using the NWChem software package.^56^ The PBEOP functional^57−59^ was selected to describe the QM region for its proven accuracy in these types of systems,^46,47,60^ the LANL2DZ^61^ basis set for Au atoms and the 6-311G(d)^62,63^ for the other atoms was used. Notice that the systems under study contain a metallic substrate and an organic layer. Both moieties should be described properly, although their nature is completely different. In general, metals are better described with GGA functionals such as PBE^57,58^ while this functional is not the best one for the description of organic molecules. As a result, a good proven compromise can be reached if the PBEOP functional is used. Moreover, the basis set is large enough to obtain accurate values of the redox properties that are being studied and, simultaneously, small enough to make the calculations computationally affordable. The addition of diffuse functions to the basis set was tested, but very similar results were obtained, so we decided not to include them to save computational cost. Finally, the aqueous solvent was modeled using the COSMO approach.^64,65^ constrained DFT^66^ was employed for the cationic calculations to constrain the positive charge in the desired fragment.

The SAM models were created using a previously established protocol, which was described in earlier studies.^47^ We have chosen the Au(100) surface; being one of the most stable surfaces of this metal, it has a work function 0.1 eV lower than the most stable Au(111)^67^ and this might have some influence on the redox properties and charge transfer mechanism. Classical MD simulations were performed using the AMBER20 software package^68,69^ to sample the conformational space of the potential energy surface of both the neutral and the cationic species of each SAM. The systems were built using AmberTools 20^70^ and several in-house developed scripts. In general terms, force field parameters for both the neutral and cationic forms of each organic molecule were developed based on QM calculations performed using the PBEOP functional (see Supporting Information). Each SAM was solvated in a tetragonal simulation box of around (41 × 41 × 45) Å^3^, which contained 1441 water molecules modeled using the TIP3P solvation model.^71^ For the SAMs that held a cationic organic molecule, a chloride anion was added to neutralize the system, and the Joung and Cheatham parameters were used to describe it.^72^

After setting up the different systems, the same dynamic protocol was applied to all of them using classical MD. It is worth to note that the motion of sulfur and gold atoms was restrained by a force constant of 50 kcal/(mol · Å^2^) throughout the protocol. The protocol began with a minimization procedure during 10000 steps, in which the steepest descent algorithm^73^ was used for the first 5000 steps, and the conjugate gradient algorithm for the last 5000 steps.^74^ Next, a constant volume (NVT) progressive heating to 300 K was carried out for 500 ps, using the Langevin thermostat to control the temperature with a collision frequency of 2 ps^–1^. Then, an additional 500 ps simulation was run at 300 K in the NVT ensemble. Following this, a 1 ns simulation was carried out in the NPT ensemble to balance the volume of the system and achieve the correct density. Finally, a 500 ns production simulation was run in the NPT ensemble with the CUDA version of pmemd. To maintain a constant pressure of 1 bar, the Berendsen barostat with anisotropic position scaling and a pressure relaxation time of 2 ps was employed. An interface in the xy plane was established to balance the pressure. During the entire protocol, the particle-mesh Ewald method^75^ with a grid spacing of 1.0 Å was used to compute the electrostatic interactions, and a 10 Å cutoff was chosen for the nonbonded interactions. The SHAKE algorithm^76−78^ was used to restrain the bonds involving hydrogen atoms, and a time step of 2 fs was used during the heating, equilibration, and production stages.

For each neutral and cationic trajectory of the SAMs, a specific number of snapshots were randomly selected from the last 350 ns of the production trajectories using the MoBioTools package.^79^ To calculate the vertical ionization energies (VIEs) of the neutral species, QM/MM/COSMO calculations were performed. The QM region was chosen to have different sizes depending on the situation described, as mentioned in the Supportiong Information. Specifically, we determined in our previous work the optimal QM region to reach a compromise between computational cost and accuracy. For these calculations, the explicit solvent molecules were removed from the snapshots and replaced by the COSMO solvation model. This choice can be justified as follows: in our previous work we performed a convergence study concerning the number of explicit water molecules that should be included to reach a constant value of the one-electron oxidation potential of these systems.^47^ However, many water molecules should be added to the QM region increasing considerably the computational cost of our protocol. To overcome that problem, some tests were conducted replacing the explicit water molecules by a continuum solvation model, which gave similar values of the redox potential to the one computed with explicit water molecules. For the cationic trajectories, the vertical attachment energies (VAEs) were computed using the same QM/MM/COSMO scheme, and by introducing constrained density functional theory for the cationic version of the SAM. All calculations were carried out using the PBEOP functional and 6-311G(d) basis set with NWChem.

In order to calculate the one-electron oxidation potential of each system, the additive scheme strategy, previously proposed,^47^ was applied (see details in the Supporting Information). In this approach, the effect of having gold atoms and additional nucleobases and linkers in the QM region is calculated in independent calculations and, therefore, it is assumed that such effects are additive and do not show cooperativity. Notice that when we state that gold atoms are included in the QM region, we only accounted for the closest four gold atoms to the organic molecule also described quantum-mechanically. This can lead to some error in the determination of the potentials but still it will provide better results than the lack of QM description. Thus, a larger number of gold atoms in the QM region would probably give rise to more reliable results. In addition, it is also important to highlight that we are not taking into account the back-polarization of the gold surface. However, all these features would imply a significant increase of the computational time and, as a result, we have to reach a compromise between accuracy and computational cost. Despite these limitations, in previous works, we have demonstrated that the inclusion of cooperativity effects to our protocol gave similar results to those obtained when this feature was neglected.^47^ Analysis of the hole distribution was carried out based on the atomic charges obtained from the QM/classical final energy calculations with PBEOP/6-311G(d). The hole distribution in the QM region allowed by constrained DFT was obtained from the differences in atomic charge between each geometry in the cationic and neutral states. Additionally, the relationship between structure and energetic terms was conducted using in-house scripts and associating the parameters for each geometry to its VIE (VAE).

Finally, we have computed atomic charges, electron density redistribution upon molecular adsorption, and binding energy for the systems under study with DFT including periodic boundary conditions. We used the VASP package^80−85^ for these simulations (see details in the Supporting Information).

## Results and Discussion

### One-Electron Oxidation Potentials: Horizontal vs Vertical Charge Transfer Mechanisms

**Figure 2:**
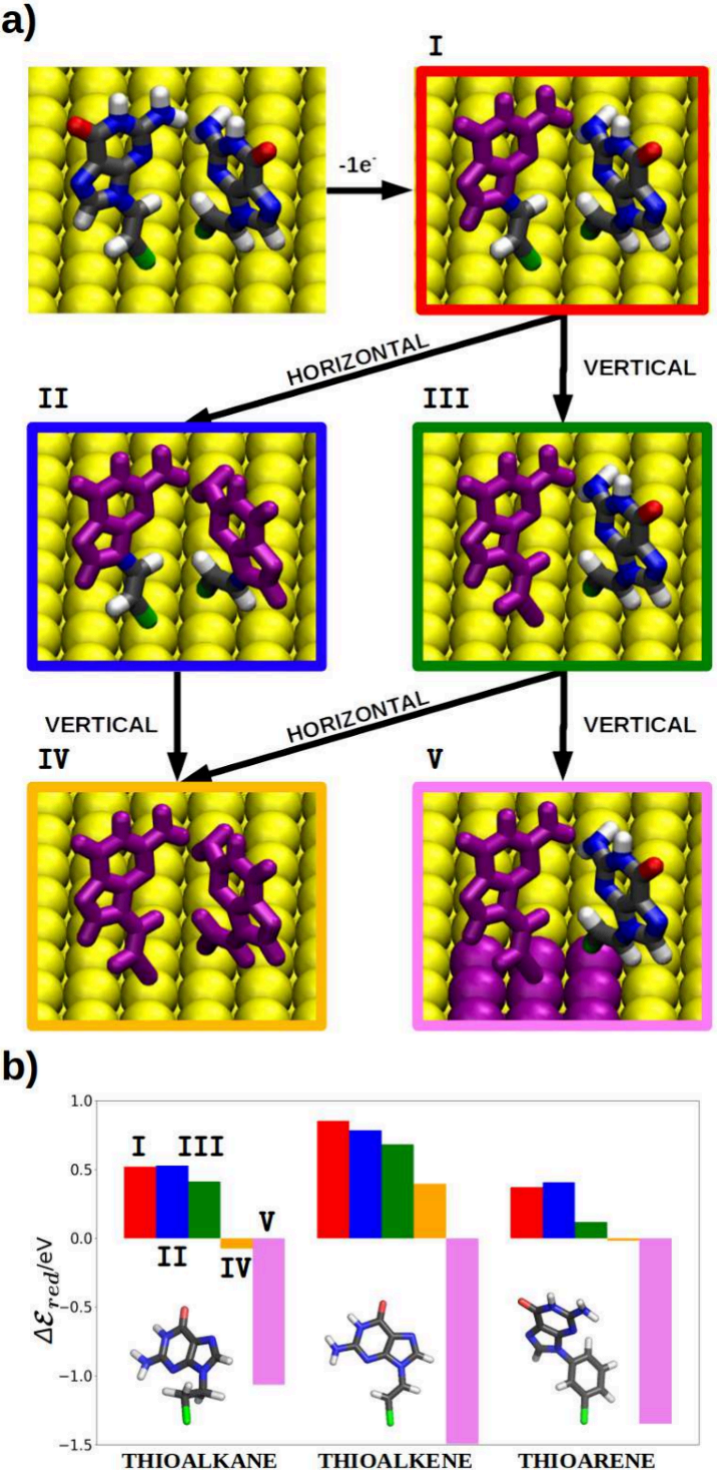
(a) Schematic representation of the different ways the charge can be delocalized either horizontally or vertically. When the SAM loses an electron from a nucleobase, the positive charge can remain within the guanine moiety (I, red box), delocalize vertically along its linker (III, green box), or reach the gold surface (V, purple box). On the other hand, the hole can delocalize among several nucleobases (II, blue box) or even among two ligands (IV, orange box). (b) One-electron oxidation potential for each situation in the three linkers considered. Color code for the atoms: C atoms in gray, N in blue, O in red, H in white, S in green.

Our discussion will begin with an overview of the different mechanisms examined in this study. Since nucleobases tend to be oxidized rather than reduced, we have only considered the case where a hole–a single positive charge–is responsible for the charge transfer. Once a nucleobase of the SAM is oxidized, giving an electron to the analyte or to another source, the former hosts a positive charge. At that point, there is a possibility that the hole may remain in the organic part of the SAM or flow toward the metallic substrate giving rise to one of the five situations displayed in Figure 2a: (I) the first step, where the charge is located on a single nucleobase of one ligand; (II) the charge is shared among other nucleobases in the SAM through horizontal charge transfer; (III) the charge is vertically transferred and is distributed along a whole ligand (nucleobase + linker); (IV) charge again hosted in the organic part, but in this case among two ligands; (V) in the vertical situation, the charge is spread on an organic ligand and at some extent is also transferred to the metal. For each situation we have computed the one-electron oxidation potential, in the three SAMs considered–with thioalkane, thialkene and thioarene as linkers – (see Δ*E*_*red*_ in Figure 2b). Based on the results, it becomes evident that charge transfer toward the gold surface occurs, irrespective of the nature of the thiolated linker. For all of them, the one-electron oxidation potential decreases drastically when the hole is allowed to access the metallic surface (see purple bars in Figure 2b). This implies that the system’s ability to donate an electron increases considerably if the hole can be partly accommodated in gold atoms.

**Figure 3:**
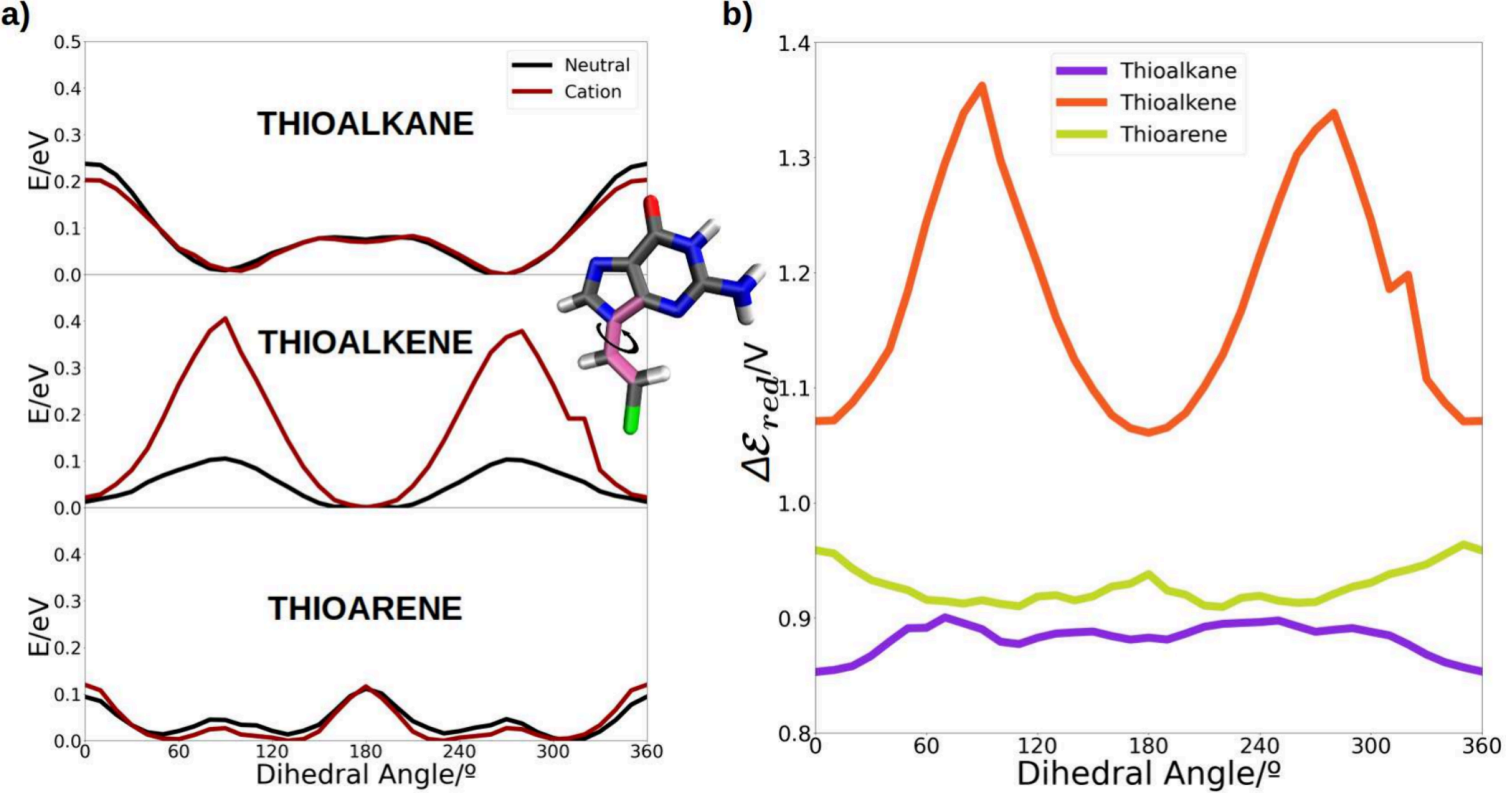
(a) Relaxed scan of the dihedral angle around the guanine-linker bond for the thioalkane, the thioalkene and the thioarene at the PBEOP/6-311G(d) level of theory on PM6 geometries. Black and dark red lines represent the relative energy profile of the dihedral angle for the neutral and cationic species of the molecule. (b) One-electron oxidation potential of the molecules along the dihedral angle.

Furthermore, when comparing the three studied systems, the relative reducing power appears to be proportional to the extent of the π-system of the molecule adsorbed on the surface. This observation can be explained by inspecting the energy profile obtained by varying the dihedral angle around the bond that connects guanine with the linker (see Figure 3). In the case of the ligand with an aliphatic linker, the linker does not contribute to the π-system of the molecule. Thus, the π-system of this ligand is restricted to guanine, which does not directly interact with the surface, and this SAM exhibits the lowest reducing power when allowing the delocalization of the positive charge to go from the ligand to the gold surface (purple bar in Figure 2b). In ascending order, in the ligand with an aromatic linker (thioarene), two π-systems can be observed: that of guanine and that of the aromatic ring. However, since the most stable configuration is nonplanar (see dihedral scan in the Figure 3), there is a decoupling of both π-systems. Thus, only the π-system of the aromatic ring directly interacts with the gold surface. The existence of this interaction may be the cause of the increased stability in hosting a positive charge in the SAM, resulting in a higher reducing power. Lastly, in the case of the thioalkene, the most stable conformation is planar, allowing the π-system to extend throughout the ligand. Therefore, this molecule possesses a larger π-system that directly interacts with the metal surface, which can explain the higher reducer character of this SAM when enabling its delocalization along a ligand and its neighboring gold atoms.

Coming back to Figure 2, two mechanisms can be identified: (i) the charge can be first delocalized among several nucleobases (horizontal delocalization) and then migrate to the surface through the linkers or (ii) it can be hosted just by one organic residue, including the linker (vertical delocalization) before reaching the metallic surface. In general terms, when the positive charge has the possibility to horizontally delocalize among several nucleobases, without considering the linkers, the one-electron oxidation potential remains constant (red and blue bars displayed in Figure 2b). Therefore, the delocalization of the hole among neighboring nucleobases does not seem to be a predominant path for charge transfer. In contrast, when the entire ligand can accommodate such a positive charge, the reducer character of the three considered systems decreases slightly (green bars in Figure 2b), supporting the idea that the hole prefers to approach the metal surface. Even more, when the delocalization of the hole between two neighboring ligands is allowed, considering also participation of the linkers in such delocalization, the potential decreases even more (orange bars in Figure 2b). Therefore, in this case, the delocalization of the charge among complete ligands does induce an increase in the reducer character of the SAMs, favoring the oxidation process. Nevertheless, the most abrupt potential change is observed when the metal substrate hosts part of the charge. This indicates that vertical charge transport along the SAM is favored.

### Charge Localization

**Figure 4:**
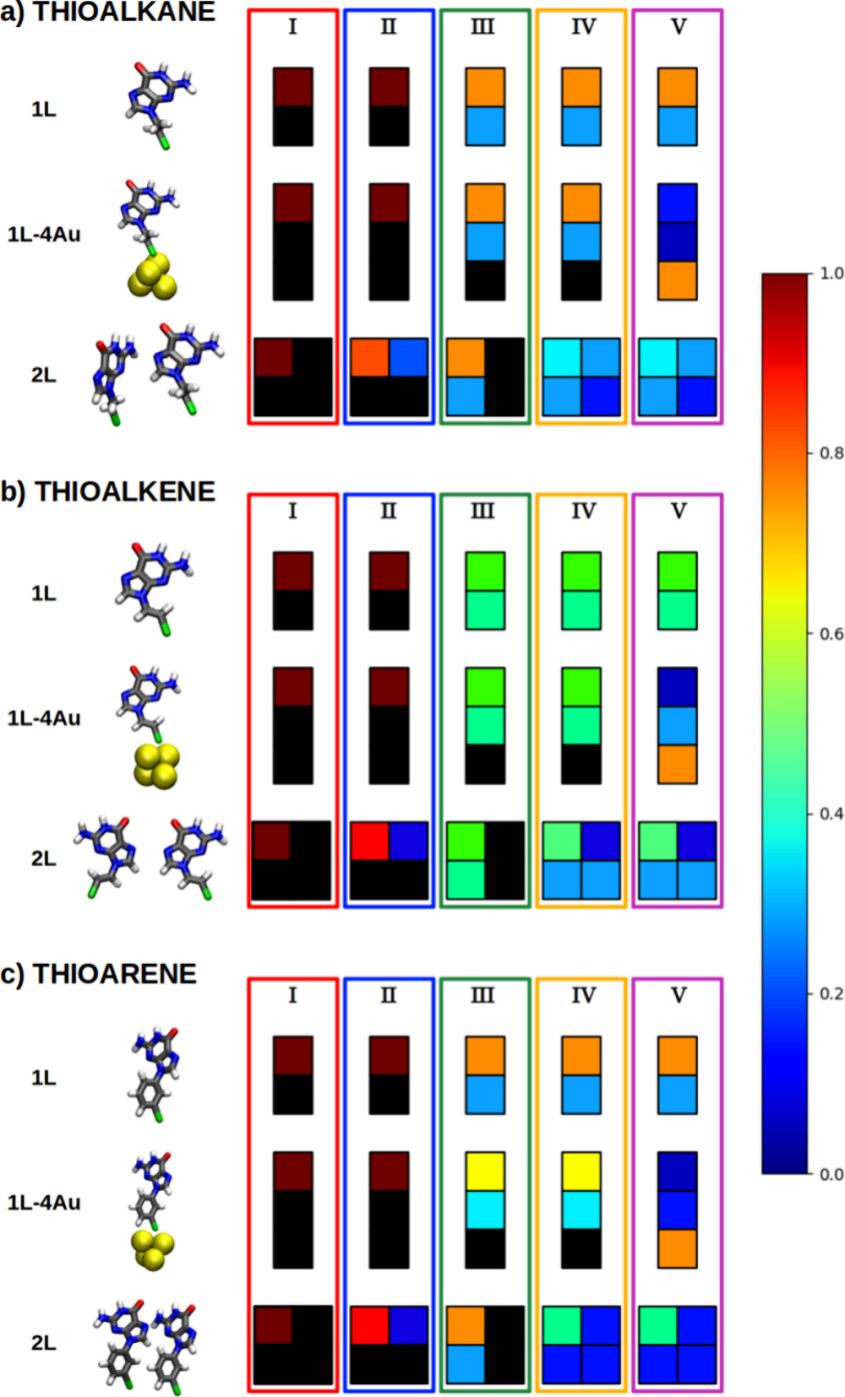
Graphical representation of the charge distribution along the different QM regions considered for the performance of the additive scheme. Each square corresponds to a nucleobase or a linker, and its color is related to the amount of charge that that moiety holds. Black squares indicate that the hole is not allowed to be held there. Additionally, the set of calculations used to calculate each pathway is surrounded by a colored line whose color points out the corresponding delocalization scenario from those considered.

To disentangle the results shown in Figure 2, an analysis of the difference in charges between the neutral and cationic species of a system with the same geometry was carried out. In other words, the spatial distribution where the hole is accommodated after the vertical ionization process was determined. Since similar results were obtained for both VAE and VIE, for simplicity only the results from the VIE will be discussed. Figure 4 shows in which components of each QM region the positive charge is stored, based on the restraints imposed with constrained DFT. Cases I to V represents the vertical and horizontal charge migration mechanisms previously discussed (see Figure 2). It should be noted that the calculation of the one-electron oxidation potential under the additive scheme was performed using three calculations: (i) the QM region consisting of the ligand (nucleobase+linker); (ii) including four gold atoms and one ligand in the QM region; (iii) the QM region consisted of two ligands. Results are given in this order in Figure 4 for each step in the mechanism and for each linker. When the hole is not allowed to be hosted in one of the components of the QM region, the corresponding box is colored in black. For a comprehensive interpretation of Figure 4, we will use case I as an example. For the first calculation, the stored charge was calculated separately for the nucleobase (upper box) and the linker (lower box); accordingly, the box representing the linker is black in those cases where the whole charge is restricted to the guanine. In the second calculation, the upper box represents the accumulated positive charge in the nucleobase, the middle box represents the accumulated charge in the linker (which is not allowed in this case), and the lower box represents the accumulated charge in the gold atoms. Finally, in the calculation involving two ligands, the two upper boxes represent the accumulated charge in each of the nucleobases of the ligands, while the lower boxes indicate the amount of hole hosted in the linkers of the respective ligands.

In those cases where the hole can only be accommodated in the nucleobase of the ligand, the charge distribution is trivial. When the delocalization is strictly vertical, the vacancy is distributed between the nucleobase and the linker along the ligand. Approximately two-thirds of that charge is stored in guanine in the cases of alkene and arene. However, this distribution is more homogeneous when it comes to the system whose ligands contain an alkene moiety (around ∼55% in the nucleobase versus ∼45% in the linker). This could be due to the conjugation of the π-systems of guanine and the linker in the case of alkene, allowing for equal delocalization of the positive charge throughout the ligand. However, in the cases of the arene and alkane linkers the situation is different. In the first case, since the ligand is not completely planar because the minimum-energy dihedral value between guanine and the linker is neither 0° nor 180°, there is no coupling between π-systems. In the second case, the alkane linker does not present aromatic moieties. Therefore, it seems that the hole prefers to stay in the nucleobase. This would explain why the potential remains constant in the cases of alkane and arene between the two situations already mentioned (see Figure 2) and yet there is a slight decrease in the potential when talking about the ligand with an alkene. Even so, we could consider that this situation, in which the hole can only be stored in a nucleobase, is equally favorable in all three cases.

Taking into account the charge distribution in the case where hole delocalization is completely vertical, i.e. when the charge can be stored in both a ligand and the metal substrate, the hole tends to be hosted approximately ∼75% in the gold atoms considered in the QM region. This supports the hypothesis that the hole tends to move toward the metal surface, leading to an increase in the reducer character of the three systems as shown in Figure 2. On the other hand, the remaining ∼25% of the positive charge is evenly distributed in the reference ligand.

If we analyze the situation where the delocalization occurs strictly horizontally in nucleobases, in all cases a clear localization of the hole in only one of the two considered nucleobases is observed. This suggests that, at least in the case of guanine, the positive charge tends to remain in only one of these moieties. This is consistent with previous articles found in the literature, where several cases have been reported in which the delocalization of a vacancy can be neglected when studying DNA strands composed of guanines in water.^86,87^ However, it seems that the interaction of a ligand with others nearby in its environment causes, by electrostatic interactions, the reduction of the one-electron oxidation potential (see Figure 2), as already demonstrated in previous works.^47^

Finally, in the case where the hole can be hosted in two ligands, including the linker in each one, a much more equitable distribution of the charge occurs. Although there is still a preference for the positive charge to reside on one of the two nucleobases, the introduction of the linker in charge delocalization causes this tendency to be blurred. Thus, by adding the ligand, the delocalization of the positive charge over the monolayer of organic ligands is increased, resulting in a drastic decrease in the one-electron oxidation potential in all three systems (see Figure 2).

Based on the results obtained so far, it could be said that the transfer of a hole from the organic monolayer to the metal substrate is quite viable and effective. This process seems to occur vertically, with some help from nearby ligands, whose linkers partially mitigate the tendency of positive charge localization in a single nucleobase. Notice that such vertical hole transfer to the metal surface is more favorable in terms of one-electron oxidation potential. This observation suggests that the transfer mechanism may involve a single ligand, where charge stabilization is achieved through electrostatic interactions with neighboring ligands, without delocalization of the hole between them. Furthermore, the distribution of the positive charge revealed that only one-third of it is located in the gold atoms, while the remaining charge is distributed mainly in one ligand, the one whose nearest gold atom also has the highest amount of charge of both.

### Structural and Energetic Analyses

**Figure 5:**
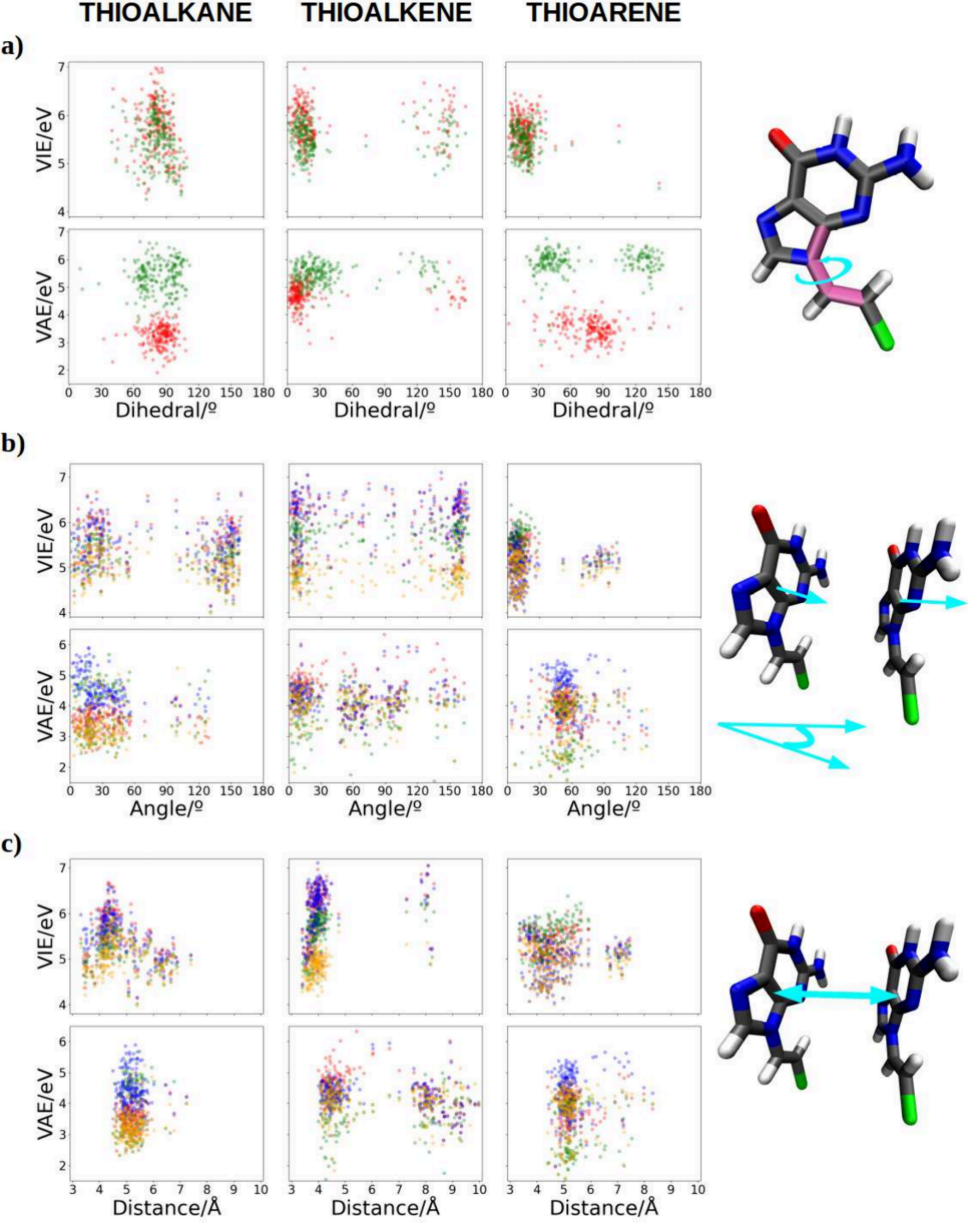
Graphical representation of the following distributions: (a) the dihedral angle, (b) the stacking angle between neighbor ligands, (c) the interligand distance. Color code: case I in red, case II in blue, case III in green, case IV in orange, and case V in purple. In the dihedral angle red corresponds to cases I and II and blue to cases III, IV, and V. A schematic representation of each parameter is represented at the right side of the plots in cyan.

So far, the behavior of the redox potential and charge distribution has been studied based on the region where the hole delocalization is allowed. However, it has not been investigated whether there is a structural component that can explain the differences observed in these values for the three analyzed systems. Additionally, the explanation for all the obtained results so far has been based on the assumption that MD follow the energy profile described in previous sections for the guanine-linker dihedral angle. This could not be the case because the dihedral parameters were taken from GAFF2 and, thus, might not reproduce the resulting potential from the QM scan. To test whether the MD and the QM scan agree, the dihedral angle has been calculated throughout all the performed dynamics (see Figure 5a). As observed, the accumulation of dihedral angle values coincides in all cases with the minima of the energy profiles shown in Figure 3a. The only case where a deviation from this profile can be observed is in the trajectory obtained from the neutral species of the SAM containing thioarenes. In this case, the range of dihedral angles is around 10–30°, while the profile predicts a minimum at 45°. However, we do not consider this deviation to be excessively significant, especially taking into account the MD simulations are close to the QM minimum. Nevertheless, except for this case, the thioalkane SAM maintains a range of dihedral angles centered at 90°, the thioalkene SAM has a range centered at 15°, and the cationic trajectory of the thioarene centers its range at an angle of 45°. Thus, it can be stated that this dihedral behaves in the MD simulations as expected from the QM calculations.

We now compare distribution of values obtained for VIEs and VAEs as a function of the dihedral angle, for calculations in which the QM region includes only the ligand, linker + nucleobase (see Figure 5a). Some general trends can be observed for the three studied systems. When the charge is delocalized throughout the ligand (green points), the VIE decreases slightly in comparison with the cases where the charge is on the guanine molecules (red points), showing a similar distribution of the dihedral angle in all cases. However, an opposite situation is observed for the VAE: it increases in ∼1–4 eV when the charge is delocalized in the whole ligand; this trend is observed in the three systems, with the increase in VAE being less pronounced in the thioalkane case. When these three ligands are free in the aqueous phase, they have similar VIEs,^47^ similar to what is observed here when they form a SAM. Therefore, it can be stated that VIE is not responsible for the changes in the redox properties of the molecules when assembled on a metal surface. However, VAE values reflect larger changes: introducing the ligands into SAM likely modifies the region corresponding to the minimum energy in the potential energy surface of cationic system but not of the neutral one, because if the last one was also modified the VIE would also change. It appears that when the charge is localized exclusively in the nucleobase, VAE values are smaller, probably due to the fact that when the electron is not allowed to be delocalized the energy released when the neutral species is formed is smaller than when the electron can be completely delocalized among both the nucleobase and the linker. Furthermore, the decrease in VAE, when the hole is localized, is more pronounced in the thioalkane and thioarene SAMs (∼3 eV). In these SAMs, it should be noted that the ligands are not planar, unlike the case of thioalkene which shows a dihedral close to 0°. It should be noted that the lowest values of the VAE are obtained by artificially constraining the charge in the nucleobase and it is, therefore, an unrealistic situation.

In order to search for further structural components able to explain energy differences between systems and delocalization trends, we evaluated the stacking angle and the distance between the ligand holding the charge and its closest ligand. These values were correlated with the computed VIEs and VAEs including different levels of charge delocalization–cases I to V (see Figure 5, pannels b and c respectively). In the neutral trajectories, there is a greater tendency for the nucleobases to align parallel compared to the cationic simulations. Thus, a higher degree of π-stacking between them is observed in the neutral species of the SAMs, with the thioalkene-based SAM standing out. The angle distribution in the thioarene-based SAM is also quite restricted to maintaining the parallel alignment of the nucleobases, although there is a small peak around 70–100° related to the π-stacking between aromatic rings of the linker. The stacking angle distribution in the thioalkane-based SAM is wider, but still maintains some π-stacking interactions, avoiding angles where the guanines are arranged perpendicular to each other. Note that when the charge can be delocalized in two ligands (case IV, orange points in the Figure 5b), lower VIEs are observed in all systems. This effect is particularly more pronounced in the thioalkene-based SAM, due to the larger π-system. This is consistent with the observed stacking angle distribution, as allowing the interaction between adjacent nucleobase π-systems makes the delocalization of the charge between them more likely, enabling a better accommodation of the positive charge, thus reducing the VIE.

When analyzing the distribution of stacking angles (Figure 5b), for the neutral species we observe distributions mainly centered at ∼0–30° for alkane and arene; the alkene exhibits a narrower angle distribution closer to a π-stacking situation. A wider angle distribution is shown by the alkane. Significant differences in the angle distribution are observed between the neutral and the cationic cases in thioalkene and thioarene SAMs. More pronounced changes are shown in the thioarene case, where a clear shift from 10° to 60° is appreciated. In the thioalkane SAM the distribution of stacking angles in the cationic species is somehow closer to the one of its neutral counterpart. Thioalkene shows an intermediate situation, with changes in the angle distributions of neutrals vs cations, but with differences not as pronounced as in the thioarene case. The VIE distributions when the charge is localized on the nucleobases (red and blue points, case I and II respectively) is typically centered in ∼6 eV (thioalkane), ∼ 6.5 eV (thioalkene) and ∼5.5 eV (thioarene). Lower VIE values when the charge is localized on the nucleobase corresponds to a higher π–stacking (angle ∼0–15°). In this context, higher VIE values of the thioalkene could arise due to the instability caused by the constraint of the charge within the nucleobase. Remember that the thioalkene moeity tends to be planar and the π-system of both the guanine and the alkene are conjugated, so that one could think that the charge will be more delocalized among the full ligand.

The distributions of the VAE values are more differentiated when the charge is localized in the nucleobase(s) or when it is delocalized in the ligand(s), with respect to the neutral scheme. This is particularly observed in the thioalkane-based SAM, with an important decrease in the VAE when the charge is delocalized in two ligands (case IV, orange points). However, charge delocalization in only one ligand (case III, green points) seems to be favored in thioarene, reducing the VAE drastically (∼2 eV). Therefore, as the cationic trajectories in the thioarene strongly deviate from the parallel arrangement of nucleobases, cases II and IV, where intermolecular delocalization is allowed, yield higher VAEs than case III (charge localized in a single-ligand). In other words, the loss of π-stacking suggests a favorable VAE toward vertical charge delocalization. In the VIE, we observe the opposite situation, i.e. close π–stacking yields higher values in case III (green points) and lower values of case IV (orange points), thus clearly favoring charge delocalization horizontally in two ligands.

On the other hand, when studying the separation between adjacent nucleobases (see Figure 5c), we observe that the distance between them slightly increases in the cationic simulations compared to their neutral counterparts, being centered in all cases at ∼5 Å. The general relative distribution of VIEs and VAEs with the distance, in the different SAMs, is similar to that of observed for the angles. Therefore, there does not seem to be a significant relationship between the distance and changes in these energy terms. Consequently, we could conclude that a certain dependence of VIEs/VAEs has been found in terms of the stacking angle between nucleobases, but not between their separation distance.

## Conclusions

In conclusion, in this work we have theoretically evaluated the one-electron oxidation potential of guanine-based SAMs adsorbed on a gold surface. We have considered different scenarios in which the created hole is transferred from an organic ligand monolayer to the metal. These scenarios have been analyzed for different monolayers in which, for each ligand, a guanine is anchored to the substrate through a linker of different nature, forming the SAM. The three analyzed systems present an alkane, alkene, or arene linker, respectively. Our results demonstrate that the most probable path is a vertical charge transfer between a ligand and the gold surface. The mechanism is favored by the electrostatic interactions that occur between ligands, stabilizing the positive charge which is mainly carried by just one ligand of the SAM. Additionally, in those SAMs where the π-system is more extensive, we observe an increase in the reducer character, which favors the transfer to the gold surface. Those SAMs with an alkene possess complete π-conjugation of the linker and guanine, resulting in the largest π-conjugated system and leading to the most efficient transfer to the substrate among the three considered systems. Although in the other two systems such conjugation is not achieved due to a torsion of the dihedral angle formed between the guanine and the linker, in the case of arene the linker itself presents a π-system that allows for more efficient transfer than in the case of alkane, whose unique π-system is reduced to that of the guanine, which is not in direct contact with the surface. We have also evaluated further structural parameters that affect the redox properties; we demonstrate a clear correlation between the stacking angle formed between neighbor nucleobases and the VIEs/VAEs values. In summary, we have obtained information about the mechanism of the charge transfer of a hole from an organic monolayer toward a metallic bulk; thus delving into the mode of operation of some technological applications of DNA, such as electrochemical molecular recognition.

## Supporting Information Available

Theoretical background of the methodology employed, protocol to obtain the parameters of the force field, additive scheme protocol, and calculations including periodic boundary conditions (PDF)
