# Supplementary material for: Charge Transfer Mechanism in Guanine-Based Self-Assembled Monolayers on a Gold Surface
Source: Langmuir. 2024 Jul 10;40(29):15129–39. doi: 10.1021/acs.langmuir.4c01512 (PMC11270990; doi:10.1021/acs.langmuir.4c01512)
Supplement: Supplementary file 1 — la4c01512_si_001.pdf [file la4c01512_si_001.pdf]

Supporting Information for "Charge Transfer mechanism in Guanine-based Self-Assembled Monolayers on a Gold Surface"

Jesús Lucia-Tamudo,[†] Juan J. Nogueira,[*,†,‡] and Sergio Díaz-Tendero[*,†,‡,¶]

†*Department of Chemistry, Universidad Autónoma de Madrid, 28049, Madrid, Spain*

‡*Institute for Advanced Research in Chemistry (IAdChem), Universidad Autónoma de Madrid, 28049 Madrid, Spain*

¶*Condensed Matter Physics Center (IFIMAC), Universidad Autónoma de Madrid, 28049 Madrid, Spain*

E-mail: juan.nogueira@uam.es; sergio.diaztendero@uam.es

Methods

One of the challenges in studying large systems is that there may be multiple local minima along the potential energy surface (PES), making it necessary to explore all possible conformations to obtain accurate values for the system's properties. This can be achieved through a sampling procedure in the theoretical model. In the case of this study, the size of the system is significantly large due to the presence of an ensemble of organic ligands forming a self-assembled monolayer (SAM) on a gold surface, and the fact that the entire system is in aqueous solution adds even more complexity. As a result, a dynamic protocol is required to accurately describe the properties of the SAM. When incorporating conformational dynamics into a model, it is often challenging to calculate the thermal correction explicitly. To

address this, an alternative method is to consider an ensemble of conformations along the potential energy surface (PES) and average the property of interest over all of them. This can provide an indirect approach to estimating the thermal correction.

For a reduction half-reaction such as the following:

$$N^{+}(aq) + e^{-}(gas) \rightarrow N(aq) \tag{1}$$

a way to compute its associated free energy is displayed in Eq. 2.

$$\Delta G_{red} = \frac{1}{2}\Big(\langle VIE\rangle_N - \langle VAE\rangle N^{+}\Big) - G(e^{-}(gas)) \tag{2}$$

This equation is derived from the Marcus theory,[1–6] which has been widely used to study the redox properties of various systems in theoretical works.[7–9] Under the assumption of a solvent response that is linear, the free energy of a reduction half-reaction can be expressed as a combination of the vertical ionization energy (VIE) and vertical attachment energy (VAE) terms. The VIE represents the energy required to remove an electron from the neutral system, while the VAE represents the energy required to add an electron to the cationic system. The phase space in which the average value of each energetic term is computed is indicated by the subscripts of the brackets in Eq. 2. The VIE is determined for the neutral system N, while the PES of the cationic system N^{+} is used as a reference for computing VAE.

To obtain the ensemble averages of VIE and VAE, classical molecular dynamics simulations are performed. The VIE and VAE values are computed for each snapshot of an arbitrarily selected ensemble of geometries using quantum mechanics/molecular mechanics or quantum mechanics/continuum approaches.[10] The target region is included in the quantum mechanics region, while the environment is described in terms of a molecular mechanics force field or a continuum solvent model. The computed VIE and VAE values are then averaged over all the snapshots.

It is important to acknowledge that there are certain limitations to using the Marcus theory. Firstly, the distributions of VIE and VAE must exhibit a Gaussian shape. Secondly, the standard deviations of VIE and VAE, denoted as σ_{VIE} and σ_{VAE}, respectively, must be equivalent. Finally, the reorganization energy λ should be the same for both the neutral and cationic species.[8] Deviations from these conditions can result in inaccuracies in the Marcus theory. However, the quadratic model proposed by Matyushov and Voth can be used as a correction for systems that do not meet these requirements.[11,12] In this study, the Marcus theory was applied with this correction for systems that did not meet the requirements for using the Marcus theory alone.

Finally, the reduction potential of a redox equation E_{red} is associated to its free energy as follows:

$$\Delta E_{red} = \frac{\Delta G_{red}}{nF} - E_{red,SHE} \tag{3}$$

where F represents the Faraday constant, n represents the number of exchanged electrons, and $E_{red,SHE}$ represents the reduction potential of a reference electrode, specifically the standard hydrogen electrode (SHE) in this case. The value of $E_{red,SHE}$ considered in this work is 4.281 V, which has been used in previous studies.[13–17] This value already includes the contribution of the electron's free energy in the gas phase $\Delta G(e^{-}(gas))$, calculated using Fermi-Dirac statistics and equal to -0.867 kcal/mol. Therefore, this value must also be added to Eq. 2.

Furthermore, to determine the one-electron oxidation potential of a system where the positive charge is concentrated in a specific fragment, certain limitations must be imposed to meet this requirement. The basis of the Constrained DFT (CDFT) technique was first introduced by Dederichs et al.[18] In this approach, Lagrange multipliers are utilized to restrict certain observables, such as charge or spin, within a specific region of the molecule of interest.[19] With CDFT, it is possible to investigate charge-restrained systems like those examined in this study.[20,21]

Parameters of the Force Fields

The following procedure was used to obtain the set of the force field (FF) parameters. First, a relaxed scan around a specific dihedral (see Fig. 3 in the main article) was performed to obtain the minimum along that dihedral coordinate, for which the parameterization was performed. That scan was performed in a first stage with the Gaussian 16 package[22] using the PM6 semiempirical level of theory.[23] In a latter stage, the electronic energy profile along the dihedral was recomputed with PBEOP/6-311G(d) using NWChem[24] to ensure consistency. Once this fact was verified, the Hessian matrix was computed for the optimized geometries obtained from static calculations in aqueous phase. The Seminario method was then used to derive bond distance and bond angle parameters for the ligands from the Hessian matrix using PBEOP/6-311G(d).[25] The GAFF model[26] was used to take the parameters for dihedral angles, improper torsions, and Lennard-Jones (LJ) non-bonded terms. However, the non-bonded parameters for the gold atoms were obtained from the literature.[27] ESP charges were computed from PBEOP/6-311G(d) calculations in aqueous phase.

Additive Scheme

Oxidation potentials have been computed using an additive scheme, that we proposed in a recent work.[28] In this formulation, the one-electron oxidation potential of the system with QM correction is obtained from the following formula:

$$\Delta E_{red,2L-4Au} = \Delta E_{red,1L} + (\Delta E_{red,1L-4Au} - \Delta E_{red,1L}) + (\Delta E_{red,2L} - \Delta E_{red,1L}) \quad (4)$$

Here, $\Delta E_{red,2L-4Au}$ is the one-electron oxidation potential with QM correction, which takes into account both the quantum effects of the gold surface and the ligands in the monolayer. This method is based on the assumption that the effects of the gold atoms and the organic environment are additive and independent of each other. Therefore, this potential is obtained

as a sum of contributions: the potential is calculated considering only one organic ligand in the QM region ($\Delta E_{red,1L}$), and the contributions of the quantum effects of gold and the organic environment are added separately. Firstly, the one-electron oxidation potential of a QM region is calculated, which includes the reference ligand from the previous calculation and the four nearest gold atoms to the sulfur of the ligand ($\Delta E_{red,1L-4Au}$). The potential of the reference ligand ($\Delta E_{red,1L}$) is then subtracted to obtain the effects of gold on the potential. Similarly, the potential of a QM region, which includes the reference ligand and the ligand closest to it, is calculated ($\Delta E_{red,2L}$). The potential of the reference ligand ($\Delta E_{red,1L}$) is again subtracted to obtain the change in potential due to the nearby ligand. Finally, these two effects are added to the potential of the reference ligand ($\Delta E_{red,1L}$) to obtain the one-electron oxidation potential with QM correction of the system ($\Delta E_{red,2L-4Au}$).

For each of the scenarios studied (see Fig. 2 in the main article) in which the charge is restricted to a certain region of the system, CDFT was used. Thus, five situations were considered for each system. In the first, the positive charge is restricted to a single nucleobase (case I in red). To this end, the three one-electron oxidation potentials required to apply the additive scheme ($\Delta E_{red,1L}$, $\Delta E_{red,1L-4Au}$, and $\Delta E_{red,2L}$) were calculated by restricting the charge to the nucleobase of the ligand. Similarly, for the case where the charge is localized in only one organic ligand of the monolayer (case III in green), including the corresponding linker, the three potentials were calculated by restricting the charge with CDFT to the full ligand. In the third case the delocalization is strictly horizontal, *i.e.*, between two nucleobases (case II in blue). To this end, $\Delta E_{red,1L}$ and $\Delta E_{red,1L-4Au}$ were calculated by restricting the charge to the nucleobase of the ligand, while for the calculation of $\Delta E_{red,2L}$ the hole was allowed to delocalize between both nucleobases. For the situation in which the charge can be delocalized among several ligands (case IV in orange), $\Delta E_{red,1L}$ and $\Delta E_{red,1L-4Au}$ were calculated by restricting the charge to the ligand, including the linker. However, for $\Delta E_{red,2L}$, the accommodation of the vacancy between two complete ligands was allowed. Finally, in the case where the hole reached the metal surface (case V in violet), for $\Delta E_{red,1L}$ the charge

was distributed among the atoms of the ligand, for $\Delta E_{red,1L-4Au}$ the charge was allowed to be in the ligand and in the gold atoms considered in the QM region, and for $\Delta E_{red,2L}$ the charge was delocalized between the two complete ligands in the QM region.

In this context, in four of these five scenarios the hole remains in the organic monolayer. In this situation, the hole can be localized exclusively in the nucleobase of a ligand (case I in red) or in a single ligand (case III in green). It can be also delocalized among multiple ligands, either exclusively between several nucleobases (case II in blue), or among complete ligands including the linker (case IV in orange). On the other hand, the hole can be transferred from the organic monolayer to the gold surface (case V in violet). The corresponding discussion is given in the main article.

Calculations including periodic boundary conditions

We have computed atomic charges, binding energies and electronic redistribution upon molecular adsorption for the studied molecules using the VASP package,[29–34] which impose periodic boundary conditions. Pseudopotentials were introduced to model the interaction between ions and valence electrons, and the wave function was described with a plane wave basis set expanded up to a cutoff of 420 eV. In all calculations we used the density functional theory within the OPTPBE functional.[35] Five Au(100) layers were modelled on different xy planes on an orthorhombic unit cell. The z direction of the cell was set to 50 Å so that we ensure that there is no interaction between different unit cells in the z direction. Then, we performed two calculations, placing the molecule adsorbed onto the surface and far from it, and we optimized the geometries in both cases in the Γ point. Once the optimizations were done, we computed the energy of the systems with a larger K-point sampling (3x3x1) to obtain more accurate energy values. Afterwards, we used the computational tools developed by the Henkelman group from the Texas University in order to compute the Bader atomic charges.[36–38]

We summarize here the obtained results:

Table 1: Bader charges.

BADER CHARGES			
	Au	Linker	Nucleobase
THIOALKANE (SAM)	72%	3%	25%
THIOALKENE (SAM)	79%	4%	17%
THIOARYLE (SAM)	75%	4%	21%
THIOALKANE	-	37%	63%
THIOALKENE	-	58%	42%
THIOARYLE	-	47%	53%

Table 2: Binding energies.

BINDING ENERGIES			
	THIOALKANE	THIOALKENE	THIOARYLE
E_{bind} (kcal/mol)	-61.34	-47.27	-48.20

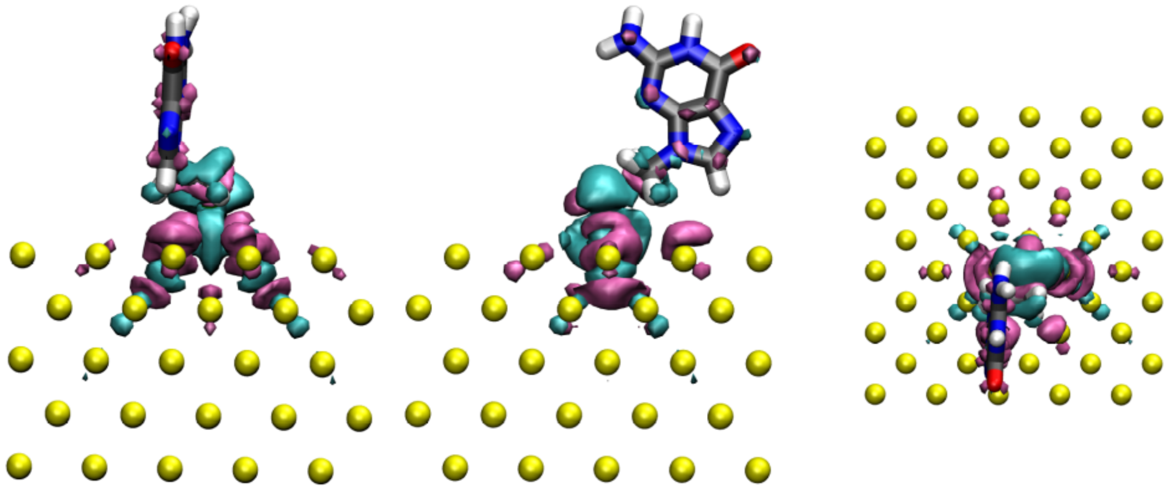

Figure S1: Schematic representation of $\Delta\rho$ of thioalkane upon adsorption. Similar results were obtained for thioalkene and thioaryle. Violet isosurfaces are associated to $\Delta\rho$ of 0.005 a.u., while cyan regions correspond to $\Delta\rho$ of -0.005 a.u.

References

(1) Marcus, R. A. On the Theory of Oxidation-Reduction Reactions Involving Electron Transfer. I. J. Chem. Phys. **1956**, 24, 966–978.

(2) Marcus, R. A. On the Theory of Oxidation-Reduction Reactions Involving Electron Transfer. II. Applications to Data on the Rates of Isotopic Exchange Reactions. J. Chem. Phys. **1957**, 26, 867–871.

(3) Marcus, R. A. On the Theory of Oxidation-Reduction Reactions Involving Electron Transfer. III. Applications to Data on the Rates of Organic Redox Reactions. J. Chem. Phys. **1957**, 26, 872–877.

(4) Marcus, R. A. On the Theory of Oxidation-Reduction Reactions Involving Electron Transfer. V. Comparison and Properties of Electrochemical and Chemical Rate Constants. J. Phys. Chem. **1963**, 67, 853–857.

(5) Marcus, R. A. On the theory of electron-transfer reactions. VI. Unified treatment for homogeneous and electrode reactions. J. Chem. Phys. **1965**, 43, 679–701.

(6) Marcus, R. A. Electrostatic Free Energy and Other Properties of States Having Nonequilibrium Polarization. I. J. Chem. Phys. **1956**, 24, 979–989.

(7) Cárdenas, G.; Marquetand, P.; Mai, S.; González, L. A Force Field for a Manganese-Vanadium Water Oxidation Catalyst: Redox Potentials in Solution as Showcase. Catalysts **2021**, 11, 493.

(8) Diamantis, P.; Tavernelli, I.; Rothlisberger, U. Redox Properties of Native and Damaged DNA from Mixed Quantum Mechanical/Molecular Mechanics Molecular Dynamics Simulations. J. Chem. Theory Comput. **2020**, 16, 6690–6701.

(9) Lucia-Tamudo, J.; Cárdenas, G.; Anguita-Ortiz, N.; Díaz-Tendero, S.; Nogueira, J. J.

Computation of Oxidation Potentials of Solvated Nucleobases by Static and Dynamic Multilayer Approaches. J. Chem. Inf. Model. **2022**, 62, 3365–3380.

(10) Senn, H. M.; Thiel, W. QM/MM Methods for Biomolecular Systems. Angew. Chem. Int. Ed. **2009**, 48, 1198–1229.

(11) Matyushov, D. V.; Voth, G. A. Modeling the free energy surfaces of electron transfer in condensed phases. J. Chem. Phys. **2000**, 113, 5413–5424.

(12) Small, D. W.; Matyushov, D. V.; Voth, G. A. The Theory of Electron Transfer Reactions: What May Be Missing? J. Am. Chem. Soc. **2003**, 125, 7470–7478.

(13) Truhlar, D. G.; Cramer, C. J.; Lewis, A.; Bumpus, J. A. Molecular Modeling of Environmentally Important Processes: Reduction Potentials. J. Chem. Educ. **2004**, 81, 596–604.

(14) Truhlar, D. G.; Cramer, C. J.; Lewis, A.; Bumpus, J. A. Erratum: Molecular modeling of environmentally important processes: Reduction potentials (Journal of Chemical Education (2004) 81 (596-604)). J. Chem. Educ. **2007**, 84, 934–934.

(15) Isse, A. A.; Gennaro, A. Absolute Potential of the Standard Hydrogen Electrode and the Problem of Interconversion of Potentials in Different Solvents. J. Phys. Chem. B **2010**, 114, 7894–7899.

(16) Kelly, C. P.; Cramer, C. J.; Truhlar, D. G. Aqueous Solvation Free Energies of Ions and Ion-Water Clusters Based on an Accurate Value for the Absolute Aqueous Solvation Free Energy of the Proton. J. Phys. Chem. B **2006**, 110, 16066–16081.

(17) Marenich, A. V.; Ho, J.; Coote, M. L.; Cramer, C. J.; Truhlar, D. G. Computational electrochemistry: prediction of liquid-phase reduction potentials. Phys. Chem. Chem. Phys. **2014**, 16, 15068–15106.

(18) Dederichs, P. H.; Blügel, S.; Zeller, R.; Akai, H. Ground States of Constrained Systems: Application to Cerium Impurities. Phys. Rev. Lett. **1984**, 53, 2512–2515.

(19) Kaduk, B.; Kowalczyk, T.; Van Voorhis, T. Constrained Density Functional Theory. Chem. Rev. **2012**, 112, 321–370.

(20) Wu, Q.; Van Voorhis, T. Constrained Density Functional Theory and Its Application in Long-Range Electron Transfer. J. Chem. Theory Comput. **2006**, 2, 765–774.

(21) Plaisance, C. P.; van Santen, R. A.; Reuter, K. Constrained-Orbital Density Functional Theory. Computational Method and Applications to Surface Chemical Processes. J. Chem. Theory Comput. **2017**, 13, 3561–3574.

(22) Frisch, M. J.; Trucks, G. W.; Schlegel, H. B.; Scuseria, G. E.; Robb, M. A.; Cheeseman, J. R.; Scalmani, G.; Barone, V.; Petersson, G. A.; Nakatsuji, H.; Li, X.; Caricato, M.; Marenich, A. V.; Bloino, J.; Janesko, B. G.; Gomperts, R.; Mennucci, B.; Hratchian, H. P.; Ortiz, J. V.; Izmaylov, A. F.; Sonnenberg, J. L.; Williams-Young, D.; Ding, F.; Lipparini, F.; Egidi, F.; Goings, J.; Peng, B.; Petrone, A.; Henderson, T.; Ranasinghe, D.; Zakrzewski, V. G.; Gao, J.; Rega, N.; Zheng, G.; Liang, W.; Hada, M.; Ehara, M.; Toyota, K.; Fukuda, R.; Hasegawa, J.; Ishida, M.; Nakajima, T.; Honda, Y.; Kitao, O.; Nakai, H.; Vreven, T.; Throssell, K.; Montgomery, J. A., Jr.; Peralta, J. E.; Ogliaro, F.; Bearpark, M. J.; Heyd, J. J.; Brothers, E. N.; Kudin, K. N.; Staroverov, V. N.; Keith, T. A.; Kobayashi, R.; Normand, J.; Raghavachari, K.; Rendell, A. P.; Burant, J. C.; Iyengar, S. S.; Tomasi, J.; Cossi, M.; Millam, J. M.; Klene, M.; Adamo, C.; Cammi, R.; Ochterski, J. W.; Martin, R. L.; Morokuma, K.; Farkas, O.; Foresman, J. B.; Fox, D. J. Gaussian 16 Revision C.01. 2016; Gaussian Inc. Wallingford CT.

(23) Stewart, J. Optimization of parameters for semiempirical methods V: Modification of

NDDO approximations and application to 70 elements. J. Mol. Model. **2008**, 13, 1173–213.

(24) Valiev, M.; Bylaska, E. J.; Govind, N.; Kowalski, K.; Straatsma, T. P.; Van Dam, H. J. J.; Wang, D.; Nieplocha, J.; Apra, E.; Windus, T. L.; de Jong, W. A. NWChem: A comprehensive and scalable open-source solution for large scale molecular simulations. Comput. Phys. Commun. **2010**, 181, 1477–1489.

(25) Seminario, J. M. Calculation of intramolecular force fields from second-derivative tensors. Int. J. Quantum Chem. **1996**, 60, 1271–1277.

(26) Wang, J.; Wolf, R. M.; Caldwell, J. W.; Kollman, P. A.; Case, D. A. Development and testing of a general amber force field. J. Comput. Chem. **2004**, 25, 1157–1174.

(27) Bhadra, P.; Siu, S. W. I. Comparison of Biomolecular Force Fields for Alkanethiol Self-Assembled Monolayer Simulations. J. Phys. Chem. C **2017**, 121, 26340–26349.

(28) Lucia-Tamudo, J.; Nogueira, J. J.; Díaz-Tendero, S. An Efficient Multilayer Approach to Model DNA-Based Nanobiosensors. J. Phys. Chem. B **2023**, 127, 1513–1525.

(29) Kresse, G.; Hafner, J. Ab initio molecular dynamics for liquid metals. Phys. Rev. B **1993**, 47, 558–561.

(30) Kresse, G.; Hafner, J. Ab initio molecular-dynamics simulation of the liquid-metal–amorphous-semiconductor transition in germanium. Phys. Rev. B **1994**, 49, 14251–14269.

(31) Kresse, G.; Hafner, J. Norm-conserving and ultrasoft pseudopotentials for first-row and transition elements. J. Phys. Condens. Mat. **1994**, 6, 8245.

(32) Kresse, G.; Furthmüller, J. Efficiency of ab-initio total energy calculations for metals and semiconductors using a plane-wave basis set. Comp. Mater. Sci. **1996**, 6, 15–50.

(33) Kresse, G.; Furthmüller, J. Efficient iterative schemes for ab initio total-energy calculations using a plane-wave basis set. Phys. Rev. B **1996**, 54, 11169–11186.

(34) Kresse, G.; Joubert, D. From ultrasoft pseudopotentials to the projector augmented-wave method. Phys. Rev. B **1999**, 59, 1758–1775.

(35) Klimeš, J.; Bowler, D. R.; Michaelides, A. Chemical accuracy for the van der Waals density functional. J. Phys. Cond. Matter **2009**, 22, 022201.

(36) Henkelman, G.; Arnaldsson, A.; Jónsson, H. A fast and robust algorithm for Bader decomposition of charge density. Comp. Mater. Sci. **2006**, 36, 354–360.

(37) Sanville, E.; Kenny, S. D.; Smith, R.; Henkelman, G. Improved grid-based algorithm for Bader charge allocation. J. Comput. Chem. **2007**, 28, 899–908.

(38) Tang, W.; Sanville, E.; Henkelman, G. A grid-based Bader analysis algorithm without lattice bias. J. Phys. Condens. Mat. **2009**, 21, 084204.
